# Bioimpedance Identifies Body Fluid Loss after Exercise in the Heat: A Pilot Study with Body Cooling

**DOI:** 10.1371/journal.pone.0109729

**Published:** 2014-10-03

**Authors:** Hannes Gatterer, Kai Schenk, Lisa Laninschegg, Philipp Schlemmer, Henry Lukaski, Martin Burtscher

**Affiliations:** 1 Department of Sport Science, University Innsbruck, Innsbruck, Austria; 2 Department of Kinesiology and Public Health Education, University of North Dakota, Grand Forks, North Dakota, United States of America; University of Palermo, Italy

## Abstract

**Purpose:**

Assessment of post-exercise changes in hydration with bioimpedance (BI) is complicated by physiological adaptations that affect resistance (R) and reactance (Xc) values. This study investigated exercise-induced changes in R and Xc, independently and in bioelectrical impedance vector analysis, when factors such as increased skin temperature and blood flow and surface electrolyte accumulation are eliminated with a cold shower.

**Methods:**

Healthy males (n = 14, 24.1±1.7 yr; height (H): 182.4±5.6 cm, body mass: 72.3±6.3 kg) exercised for 1 hr at a self-rated intensity (15 BORG) in an environmental chamber (33°C and 50% relative humidity), then had a cold shower (15 min). Before the run BI, body mass, hematocrit and Posm were measured. After the shower body mass was measured; BI measurements were performed continuously every 20 minutes until R reached a stable level, then hematocrit and Posm were measured again.

**Results:**

Compared to pre-trial measurements body mass decreased after the run and Posm, Hct, R/H and Xc/H increased (p<0.05) with a corresponding lengthening of the impedance vector along the major axis of the tolerance ellipse (p<0.001). Changes in Posm were negatively related to changes in body mass (r = −0.564, p = 0.036) and changes in Xc/H (r = −0.577, p = 0.041).

**Conclusions:**

Present findings showed that after a bout of exercise-induced dehydration followed by cold shower the impedance vector lengthened that indicates fluid loss. Additionally, BI values might be useful to evaluate fluid shifts between compartments as lower intracellular fluid loss (changed Xc/R) indicated greater Posm increase.

## Introduction

Dehydration is recognized to impair both, physical and mental performance, particularly if fluid loss exceeds 2% of body mass [1]. Body fluid monitoring may permit adequate recommendation of increased fluid intake and thus limit deleterious effects of marked dehydration. However, there is no consensus on a single method to monitor hydration status, as no single assessment method/technique seems valid during daily activities of athletes [2]. Therefore, new methods/techniques that assess hydration in real time and in a precise, accurate, reliable, non-invasive, portable, inexpensive, safe and simple way, are needed [2]. The bioelectrical impedance vector analysis (BIVA), described in detail by Piccoli et al. [3] and Lukaski et al. [4], could fill this gap. By using BIVA, it is possible to express changes of hydration status of soft tissues, by solely considering impedance components (i.e. resistance and reactance) independently of regression predictions of fluid volumes or assumptions about the constant chemical composition of the fat-free body [3], [4]. As such, the major weaknesses described for the bioelectrical impedance analysis (BIA), i.e. dependency on equations and euhydration status could be eliminated [5]. The main problems associated with use of bioimpedance (BI) after exercise in the heat to assess hydration status include the increased cutaneous blood flow and temperature as well as skin electrolyte accumulation, which are known to largely affect BI measurements [6]–[8]. Cleansing the skin with cold water application after exercise, which athletes use as a recovery strategy [9], could help to overcome the artifactual effects of these physiological responses of exercise on BI measurements. We hypothesized that elimination of the confounding effects of increased skin blood flow and concurrent electrolyte accretion on BI parameters in response to exercise in the heat by cold shower application after an exercise bout will yield BI values that reflect changes in hydration status. Therefore, the present study aims were to (1) determine whether BI changes are useful to indicate fluid loss when exercise and heat induced increases in cutaneous blood flow and skin temperature were reduced by cold shower application and (2) compare impedance vector shifts with conventional hydration markers [2].

## Materials and Methods

### Participants

Sixteen healthy, well trained male adults were recruited for the study; 14 men (age: 24.1±1.7 yr, height: 182.4±5.6 cm, body mass: 72.3±6.3 kg) completed the study protocol. Two participants dropped out because they did not tolerate the cold shower. All participants were informed about the study goals and procedures and gave written informed consent to participate. The study was approved by the Institutional Review Board of the Department of Sport Science of the University of Innsbruck.

### Procedures

The day before the investigation participants were instructed to drink 3.0 L fluid (non-alcoholic beverages) over 24 h (2.0 L should be consumed between 6 and 10 pm) in addition to habitual occidental dietary practices. From 10 pm until the start of the testing (8 am the following day), no further fluid and food intake was allowed. This procedure should induce euhydration levels before the start of the testing [10].

When presenting to the laboratory, participants emptied their bladder, and then underwent a series of pre-exercise measurements including BIA, skin temperature and body mass, and determinations of hematocrit (Hct) and plasma osmolality (Posm). Whole body BIA (BIA 101 Anniversary AKERN/RJL Systems; Florence, Italy) was performed using an alternating sinusoidal electric current of 400 µA at an operating frequency of 50 kHz. The device was calibrated every morning using the standard control circuit supplied by the manufacturer with a known impedance [resistance (R)  = 380 Ω; reactance (Xc)  = 47 Ω). The accuracy of the device was 1% for R and 1% for Xc. For the BI measurement each participant was supine with limbs slightly spread apart from the body. Electrodes (BIATRODES Akern Srl; Florence, Italy) were placed on the right side at metacarpal and metatarsal sites of the right wrist and ankle [11]. The sites where electrodes were placed were marked with a waterproof pen. BI analyses were done according to the BIVA method [3]. Measurements of R and Xc were standardized by the height of subjects (i.e., R/H and Xc/H) and were expressed in ohm/m. The combination of R (i.e., opposition to the flow of an alternating current through intra- and extracellular ionic solution) and Xc (i.e., capacitive component of tissue interfaces, cell membranes and organelles) yields an impedance vector (geometric relationship  =  arc tangent of Xc/R expressed in degrees) [4]. The length of the vector is inversely related to TBW [4] and was calculated as the hypotenuses of individual impedance values [12]. This approach enables the classification of tissue hydration status and body cell mass by solely considering impedance components relative to a healthy gender matched population [4]. Skin temperature was measured using infrared thermometer (Mini Flash, TFA Dostmann GmbH & Co, Wertheim, Germany) at the back of the hand and foot in between the site where BIA electrodes were placed. Body mass was measured to the nearest 0.01 kg (DS150K1, Kern, Germany) with participants wearing swimming suits and body height was measured to the nearest 0.5 cm. Hct was measured from a capillary blood sample taken from the fingertip (Lange LP20, Dr. Lange, Berlin, Germany). For the determination of Posm, 250 µl of capillary blood (fingertip) was collected with a microvette system (Microvette CB300, Lithium heparin, Sarstedt, Germany). Blood was immediately centrifuged and Posm was determined in replicate by freezing point depression method (Fiske Micro-Osmometer, model 110; Fiske, Norwood, MA). Every morning before starting the trials the device was calibrated with two different standard solutions (50 and 850 mOsm/kg). After the calibration reference measurements were performed with a 290 mOsm/kg reference solution. The mean ± SD of these measurements were 288.5±0.8 mOsm/kg (expected range 288–292 mOsm/kg). The coefficient of variation (CV) determined from 12 consecutive measurements was 0.4%.

The exercise dehydration session was conducted in an environmental chamber set at 33°C and 50% relative humidity. Participants exercised for 1 hr at a self-rated intensity corresponding to a score of 15 (i.e. heavy work) on the BORG scale [13].

After the dehydration session, participants were instructed to take a cold shower (as cold as tolerable) for 15 min to reduce cutaneous blood flow and temperature and remove accumulated electrolytes. As reviewed by Versey et al., 15 min of body cooling is within the time frame (i.e. 5–15 min) regularly applied in cold water application settings [14]. After the shower and drying with a towel, the same measurements were performed as described in detail previously. The order of the measurements was as follows: body weight was measured once and whole-body BI measurements were performed every 20 min until two consecutive post shower R measurements did not differ more than 3 Ω which is within the accuracy of the device for our population (5.2 Ω). Electrodes were placed at the site marked by pen and were left until the end of the trial. Skin temperature, as a surrogate for cutaneous blood flow [7], was measured 3 times after the shower (last measurement approximately 80 minutes after the shower) concurrently with BI measurements. After achieving stable BI values, capillary blood samples for the determination of Hct and Posm were obtained and participants were released from the study. During all measurements, participants were seated in a thermal neutral room and were not allowed to drink or eat.

### Statistical analysis

Data analyses were performed using the SPSS statistical-software package (PASW Statistic 21). For analysis of the bioelectrical impedance data only raw values, i.e., R and Xc values and the impedance vector (calculated as the hypotenuses of individual R and Xc values) were used [4], [12]. Paired student t-tests were used to analyze changes from before to after the trial (e.g., stable R and Xc values). Paired one sample Hotelling's T^2^ test were used to calculate significant vector displacements. A correlation analysis was performed for changes of BI values and vector shifts (from before to after the stabilization) and changes in body mass, Posm and Hct. Changes (Δ) were calculated as post minus pre values. Values are presented as means ± standard deviation (SD). The level of significance was set at p<0.05.

## Results

**Figure 1:**
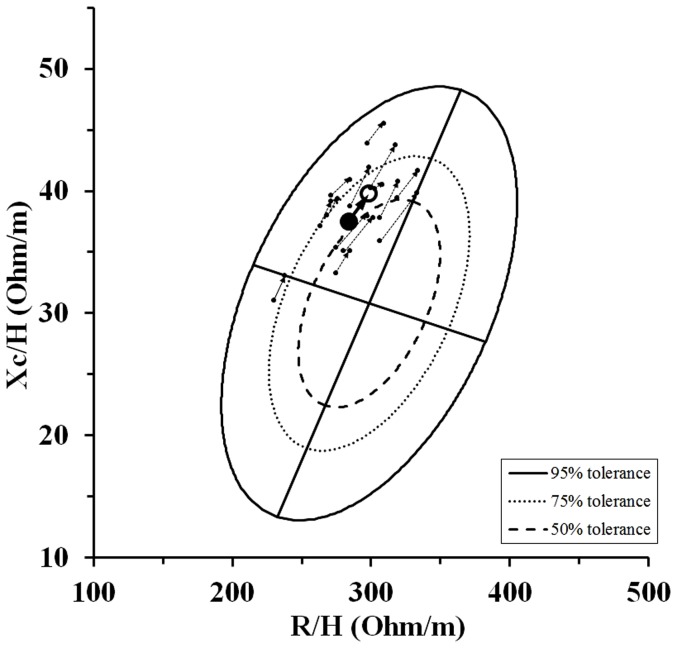
Mean (bold arrow) and single (thin dotted arrows) impedance vector shifts (from before to after the run) plotted on the 50%, 75% and 95% tolerance ellipse of the general Italian healthy male population [15].

**Figure 2:**
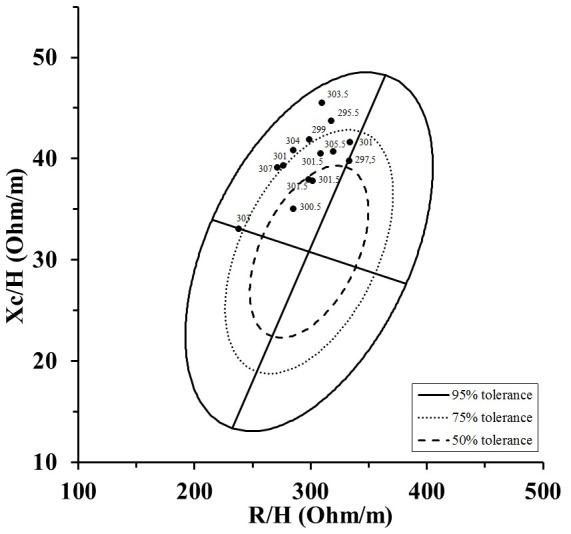
Individual vectors after the dehydration session and the corresponding Posm value.

All participants completed the 60 min exercise session without any problems. After the bout of exercise and shower, significant changes of body mass, Posm, Hct and BI values occurred (Table 1). Body weight decreased 2.1±0.4%. Mean time required until BI values after the shower reached a stable level was 75±23 min. The group impedance vector lengthened significantly (p<0.001) after exercise in the heat and the individual impedance vectors (Figure 1) extended along the major axis of the tolerance ellipse of the healthy, male reference population [15]. Figure 2 shows individual vectors after the dehydration session and the corresponding Posm values. The Δ Posm was negatively related to Δ body mass (r = −0.564, p = 0.036) and Δ Xc/H (r = −0.577, p = 0.041).

**Table 1:** Measured parameters before and after the exercise dehydration plus cooling session.

	pre	post	p-value
body mass (kg)	72.29±6.34	70.78±6.18	<0.001
Posm (mOsm/kg)	295.5±4.4	301.6±3.0	<0.001
Hct (g/dl)	45.7±3.5	47.4±2.7	0.033
R/H (Ω/m)	284.1±23.0	298.0±26.0	<0.001
Xc/H (Ω/m)	37.5±3.3	39.8±3.2	<0.001
vector length	286.6±23.1	300.6±26.0	<0.001
skin temperature (°C)	29.3±1.1	28.8±1.3	0.105

Plasma osmolarity (Posm), resistance divided by body height (R/H), reactance divided by body height (Xc/H).

## Discussion

Main findings of the present study are that, after exercise-induced dehydration, impedance vectors significantly lengthened along the major axis of the tolerance ellipse in conformity with fluid loss (decreases in total body water, Figure 1). Furthermore, changes in Xc/H were significantly negatively related to Posm changes, likely indicating fluid shifts between intracellular and extracellular compartments.

A broad number of studies investigated the validity of BI measurements to assess changes in hydration status in healthy people with ambiguous outcomes [4], [16]–[18]. Data indicate that BI adequately assesses alterations in hydration status when changes were induced passively (e.g. by fasting) [16] or BI measurements were performed hours after an exercise-induced dehydration session [17]. On the contrary BI failed to identify changes in hydration status when measured acutely after exercise and/or heat-induced dehydration [18]. Acute changes in cutaneous blood flow and temperature as well as skin electrolyte accumulation after exercise and heat application are well known to directly affect BI measurements [6]–[8] and could explain these findings. To the best of our knowledge, this is the first study investigating the applicability of the BI and the BIVA graph within a setting that uses cold shower to lessen or even abolish the influence of the aforementioned factors on BI after exercise induced dehydration.

Present study demonstrates that the BIVA graph reflects fluid loss after exercise within this specific setting. The lengthening of the vector along the major axis of the tolerance ellipse (Figure 1) indicates fluid loss [3], [4] and is supported by the finding that the length of the vector is inversely related to TBW [4]. However, body mass changes did not show any correlation to changes in BI values (i.e. Δ vector length, Δ R/H and Δ Xc/H). This is in contrast to other studies where significant correlations were found for BI and body mass changes when hydration changes were induced passively and/or chronically [19], [20]. One explanation could be that the criterion for stable BI values, even though the established 3 Ω differences are within the accuracy of the device, was not adequately set in present study and further R/H and Xc/H changes were unnoticed. Conversely also body mass measurements might have led to erroneous body fluid balance estimates. Even though body changes are usually considered to be useful in detecting short term body hydration changes [21], several exercise-related factors, such as sweat rate, respiratory water loss and oxidative water production may lead to substantial body mass loss without an effective net negative fluid balance [22]. Beside body weight changes, Posm is proposed as an adequate dehydration marker [10], [23] despite controversy regarding its specificity and sensitivity to detect dehydration [2], [24].

We observed that changes in Posm were correlated to changes in body mass and Xc/H (Table 1). Whereas the relationship between exercise-induced loss of body mass (due to water loss) and changes of Posm is well described in literature [2], the finding of the observed relationship between changes in Posm and Xc/H is novel. As Xc/H reflects intracellular fluid content this relationship indicates that lower intracellular fluid losses mean greater Posm increases. Accordingly Armstrong et al. stated that exercise might induce increases in intracellular osmolality and extracellular fluid tonicity leading to water shifts between these two compartments [24].

Within the clinical setting reference intervals for the BIVA graph exist that allow classification of owns hydration status. It was described that vectors within the 50% tolerance ellipse reflect normal hydration, whereas lengthened vectors out of the upper pole of the 50% and 75% tolerance ellipses indicate mild and severe dehydration, respectively [4]. When using the Posm threshold for dehydration assessment of 301±5 mOsm/kg, as suggested by Cheuvront et al [10], the tolerance ellipses of the general Italian healthy male population [14] were not suitable for the classification of the hydration status (Figure 2). However, it has to be mentioned that the Posm threshold is not generally considered the gold standard for dehydration assessment [2], [24]. Furthermore population specific reference ellipses might be necessary when performing such analyses as significant differences might exist between [25] and even within different sport disciplines [26]. Participants of present investigation had different sports background. Therefore different population specific reference ellipses would have been required which are not available so far.

Some issues need to be mentioned when interpreting the present results. Participants were advised to drink 3.0 L fluid the day before the trial. However when reporting to the laboratory participants were fasting for 10 hours and pre-trial values of Posm and BIVA indicated some degree of dehydration [10], [27]. Even though Cheuvront et al. [10] used this protocol to achieve a euhydrated state we cannot exclude that the 10 hours fasting might have caused mild dehydration which might have influenced outcomes. Furthermore the water temperature of the shower was not standardized and might have led to different degrees of cooling-down and thus reductions of cutaneous blood flow and temperature. Even though skin temperature was controlled in present study cutaneous blood flow was not. Cutaneous blood flow could be one factor explaining the different time courses required to achieve stable R levels post shower between subjects. Nonetheless, this procedure should have been adequate as the goal of the present investigation was not to establish the time course but to show if BIVA is able to indicate fluid losses after exercising in the heat. However, as in practice the time course may be of importance further studies using standardized protocols of cold water application with different duration and temperatures are needed. Additionally in further studies a range of different dehydration levels should be induced to establish whether the degree of vector lengthening reflects the degree of body fluid changes.

## Conclusions

In conclusion this study demonstrated that BIVA changes convincingly mirrors water loss within an exercise and heat induced fluid loss trial. Additionally Δ Xc/H values, reflecting changes in intracellular fluid content, might be useful to evaluate fluid shifts between compartments. However more studies are needed to establish if BIVA can be considered a reliable method for monitoring hydration status.
